# Ambulance Nurses’ Experience of a Simulation Exercise Concerning Intimate Partner Violence

**DOI:** 10.1111/inr.70030

**Published:** 2025-05-16

**Authors:** Edberg Matei Emma, Johansson Jeanette, Sjövall Katarina

**Affiliations:** ^1^ Faculty of Health Sciences Kristianstad University Kristianstad Sweden; ^2^ Faculty of Medicine Lund University Lund Sweden

**Keywords:** ambulance, experience, intimate partner violence, nurses, qualitative research, simulation

## Abstract

**Aim:**

To qualitatively assess an intervention for ambulance nurses, including simulation training concerning intimate partner violence (IPV), and to describe the ambulance nurses’ experiences of their work after the intervention.

**Background:**

IPV is a common public health issue with serious consequences from individual and societal perspectives. Previous research indicates that deficits in knowledge, preparedness, confidence, and clinical skills are barriers for nurses in detecting IPV. Ambulance nurses have a unique opportunity to identify IPV as they often encounter patients in their homes. However, there is limited research on the impact of simulation for training ambulance nurses to improve their preparedness and competence to respond to IPV.

**Design:**

An intervention study using qualitative semi‐structured interviews that were analysed using content analysis.

**Method:**

Simulation pedagogy was used for training ambulance nurses (*n* = 7) in how to deal with women suspected of having been exposed to IPV. Qualitative interviews were conducted to collect data about the ambulance nurses’ experiences with the simulation and about their work in relation to IPV after the simulation. The interviews were analysed using content analysis.

**Results:**

The results are presented through four categories and eight subcategories. The categories are: Simulation provided new insights, Simulation was like encountering a real patient, Simulation changed my way of working, and Simulation increased awareness of the importance of teamwork.

**Conclusion:**

The participants experienced increased confidence in asking about exposure to violence, resulting in more patients being asked about exposure to IPV after the simulation training. Although this was a small study, the results showed the importance of training in a realistic context and the value of interaction among participating ambulance nurses. Simulation addressing IPV can open new doors by providing new knowledge and by allowing participants to observe each other.

**Implications for Nursing Practice:**

The findings from this study might also be applicable to nurses in other contexts, such as district nurses or nurses in home‐based care work. Training with simulations can increase knowledge about IPV and provide practical strategies for how, when, and where to address the issue of IPV. This can lead to a higher likelihood of identifying and providing support to individuals experiencing IPV.

## 1 Introduction

It is reported that around 30% of all women in the world today at some point in their lives have experienced physical and/or sexual abuse (WHO 2017). Intimate partner violence (IPV) affects all dimensions of women's health; physical, psychological, social, and existential. In Sweden (about 10 million inhabitants), it is estimated that one woman every third week dies because of IPV. Death investigations show that victims and perpetrators have often had contact with healthcare close to the time of the woman's death (Swedish National Board of Health and Welfare 2022). Also, women who screen positive for IPV in the emergency department are at risk of being subjected to abuse in the subsequent period (Ahmad et al. 2017). Thus, healthcare professionals need to be prepared to encounter and support women subjected to IPV (Miller et al. 2021) as well as be mediators to help the woman access further assistance (Miller et al. 2021; Purwaningtyas et al. 2019).

### 1.1 Background

Ambulance nurses often meet their patients in their home environment and thus have a unique opportunity to see signs and make observations that the patients do not talk about (Swedish National Board of Health and Welfare 2023). In Sweden, ambulance nurses are essential in the prehospital emergency care system, providing advanced medical treatment at the scene of emergencies. Since 2005, regulations have required every ambulance to have at least one registered nurse qualified to administer medications and perform complex procedures​ (Swedish National Board of Health and Welfare 2023; Lindström et al. 2015). Ambulance nurses often receive specialised training, enabling them to manage acute conditions, make clinical assessments, and deliver critical care such as resuscitation. They play a key role in determining whether patients need hospital transport or can be treated on‐site, ensuring efficient use of emergency resources and improving patient outcomes (Swedish National Board of Health and Welfare 2023; Lindström et al. 2015). In cases when the patient is taken to hospital by ambulance, the nurse is alone with the patient, which can facilitate screening for IPV.

Previous research has shown that women wanted healthcare professionals to ask them about their exposure to violence. When screening for IPV was used, it was perceived to make a significant difference to victims of IPV (Price and Couch 2023; Ahmad et al. 2017). In Sweden, the nurse should ask about IPV whenever there is a suspicion about exposure to violence. All healthcare departments are required to have prepared guidelines for staff to be able to handle meetings with a person who is or has been subjected to violence. In some clinical areas, the question must be put to all patients, regardless of suspicion (e.g. maternal healthcare). If the question is asked routinely, with all patients, the risk of professionals’ attitudes and prejudices being an obstacle is minimised (WHO 2013; Sprague et al. 2012).

Despite the possibilities for screening and clear guidelines for when, where, and how nurses should ask about IPV, several barriers to asking the question have been described (Öhman et al. 2024; Sundborg et al. 2015). Previous research clearly indicates a lack of knowledge as a significant explanation for not asking the question (Alhalal 2020; Aregger Lundh et al. 2022; Withiel et al. 2022). Lack of knowledge and practice among nurses results in low confidence and the feeling of not being competent to ask women about IPV (Alshammari et al. 2023; Withiel et al. 2021; Alhalal 2020). Facilitating factors for asking about IPV include recognising signs of IPV and having strategies for asking about it (Öhman et al. 2024; Ahmad et al. 2017; Sundborg et al. 2015). Increased knowledge, along with practice in asking questions about exposure to violence, gives nurses increased preparedness and competence in screening for IPV (NCK 2022; Ahmad et al. 2017). Thus, continuous education and training for nurses in encountering women exposed to IPV must focus on increasing the knowledge and training.

To ensure training for exposure to complex clinical situations occurs in a supportive learning environment, simulation is used in many nursing education programmes (Alharbi et al. 2024). The learning method allows participants to practice in real nursing situations. That means they have opportunities to practise specific scenarios in an authentic and safe learning environment without the risk of harming any patient (Alharbi et al. 2024). Simulation creates opportunities for acquiring experienced‐based learning, increasing clinical knowledge, developing teamwork, and improving communication skills within a team (Alharbi et al. 2024; Allison et al. 2023). During simulations, participants are encouraged to make their own decisions and critically review their own efforts based on what worked well and not so well. At the end of a simulation session, debriefing is used to give participants the opportunity to reflect upon their learning. Debriefing to integrate learning in simulation is said to be critical for experiential learning (Allison et al. 2023).

Previous research on the simulation of IPV has focused mainly on nursing students. Interactive and practice‐focused learning interventions have been shown to produce the best results in health education about gender‐based violence (Sammut et al. 2021). Nursing students undergoing simulation training on IPV experienced an increased understanding of how to identify and support potential victims of IPV (Lee and Lee 2022; Cunningham et al. 2020; Ross et al. 2020). The simulation training also helped give the students greater confidence in practising advocacy for vulnerable persons (Ross et al. 2020; Johnson and Montgomery 2017).

Fewer studies have focused on clinically active nurses, but results indicate the same effect as for students. Tailored practice through multidimensional, interactive training (Withiel et al. 2023) or simulation including reflection has been shown to improve nurses’ clinical skills and competence in several areas (O'Rourke et al. 2023). Regular training in the context of ambulance healthcare has been shown to increase nurses’ confidence in clinical assessment and decisions, thereby preparing them mentally for the unexpected (Andersson et al. 2019). However, providing training about IPV presents challenges in ensuring it is both in‐depth and relevant to the context in which the nurse is operating (Allison et al. 2023; Withiel et al. 2023; Hinsliff‐Smith and McGarry 2017). Nurses in ambulance healthcare have no predetermined work environment; they constantly need to adapt to complex healthcare situations. Therefore, it is crucial that the training they are offered reflects the complexity they face clinically (Wallin et al. 2021). In this study, we aimed to qualitatively assess an intervention for ambulance nurses, including simulation training on IPV, and to describe their experiences of their work after completing the intervention.

## 2 Method

### 2.1 Design

**FIGURE 1:**
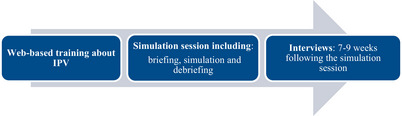
Flowchart for intervention (IPV‐education) and follow‐up.

This study used qualitative semi‐structured interviews analysed using content analysis. The study was carried out in three steps (Figure 1). Participants from an ambulance station first completed a web‐based education programme offered and developed by *the National Centre for Knowledge on Men's Violence Against Women* (Nationellt centre för kvinnofrid, NCK). The purpose was to deepen participants’ knowledge about IPV. This was followed by a simulation session with a focus on IPV, including briefing, simulation, and debriefing. The simulation was focused on meeting individuals subjected to violence and asking questions about their exposure to violence. Seven to nine weeks after the simulation, the participants were interviewed about their experiences of the simulation and how they experienced their work after it.

### 2.2 Study Setting and Recruitment

A consecutive sample was used. Information about the study was given to all nurses working at an ambulance department in the southern part of Sweden. A gatekeeper was used to inform nurses working at the ambulance station about the aim of the study, the intervention, and the following interview. The gatekeeper was a research nurse at the same unit and did not participate in the study. Information was given at a workplace meeting. Nurses interested in participating then contacted the first two authors.

Eight ambulance nurses agreed to participate, but one of them was not able to participate in the simulation session. The intervention and the following interviews were carried out between March and June 2023. All participants were familiar with simulation as a pedagogical method. All participants had former experience of meeting patients subjected to IPV. The demographic data of the participants are presented in Table 1.

**TABLE 1:** Demographic data of participants (*n* = 7).

Gender (*n* = 7)	
Female	6
Male	1
Age (years)	31–53 (mean 43)
Working as a nurse (years)	5–28 (mean 15.5)
Working as an ambulance nurse (years)	1–20 (mean 7.7)

### 2.3 The Intervention

Participants started by completing a web‐based course about IPV, developed and offered by NCK. The course covers key topics such as the societal responsibility in responding to violence, effective approaches for engaging with individuals affected by violence, and the specific challenges faced by vulnerable groups. It takes about three hours to complete. The web course is designed for professionals in social care, healthcare, education, law, and other related fields, as well as for the public. It aims to enhance the competencies of participants in understanding and addressing violence against women and children, including its causes, mechanisms, and consequences.

**FIGURE 2:**
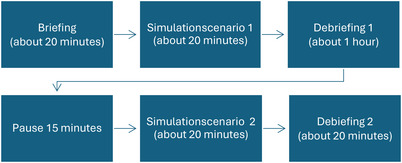
Flowchart for a simulation session.

Four participants attended first simulation session, three attended the second session. The simulation session lasted about three hours and included briefing, debriefing, and two different scenarios (Figure 2). Participants acted during one of the sessions and observed in the other.

Two instructors conducted simulation sessions, both trained in simulation pedagogy. They were not known to the participants. The instructors took turns acting as the patient in the two scenarios. The scenarios were developed to be specific for the ambulance (home) context. Fictitious patient cases were used, and make‐up was employed to illustrate physical injuries such as head injury/concussions.

The first scenario is about a young woman whose parents have called the ambulance. She has visible choke marks on her throat and her arms. She has been subjected to physical and psychological violence by her male partner and has also been sexually abused. The scenario starts with the young woman and the ambulance nurse in the ambulance. The woman says she feels dizzy as she has hit her head. The second scenario is about a middle‐aged woman who has called the ambulance herself. She has suicidal ideation and tells the ambulance nurse she was going to hang herself but she hesitated. She has been subjected to violence by her female partner, and she has visible injuries to her face and throat. Apart from physical violence, there is also psychological violence. The scenario starts with the woman and ambulance nurses in her bedroom.

### 2.4 Data Collection

Interviews were conducted 7–9 weeks after the intervention to get a deeper understanding of participants’ experiences of the simulation and if and how their daily work was affected. A pilot interview was performed before the start of the study to test the questions, the recorder, and to practice the role of interviewer. The interviews were conducted face to face or online according to the participants’ wishes. Six interviews were conducted at the ambulance station, and one interview was conducted online with the participant at home. Both authors participated in the interviews, one asking all the questions and the other one listening, observing, and taking notes. An interview guide with open‐ended questions was used. In the opening question participants were asked to describe how they experienced the simulation and its different parts and about feelings that occurred. They were then asked to say whether the simulation had affected their daily work and, if so, how. Follow‐up questions were asked to further develop and deepen participants’ narratives. The interviews lasted between 18 and 34 minutes. All except for one interview were recorded and transcribed verbatim by the first two authors. One participant did not want the interview to be recorded. On this occasion, both interviewers took notes during the interview. Directly after the interview, notes were compared and compiled.

### 2.5 Data Analysis

To describe and to get an understanding of the central themes that represent what participants experience and relate to, conventional content analysis was used to analyse the verbatim interview transcripts (Hsieh and Shannon 2005). The analysis focused on identifying similarities and differences and on describing variations. The analysis was performed by the two first authors and the process included five steps. In the *first step*, the verbatim interview transcripts were carefully read by both first authors. After the reading, the impressions gained from reading the text were discussed. The discussion was recorded for later use if necessary. In the *second step*, meaning units related to the aim of the study were identified and highlighted in the text, at first separately by the two authors and then together. In the *third step*, the meaning units were condensed and then coded according to what they expressed and to their meaning. In the *fourth step*, codes with similar meanings were categorised into clusters with similar meanings. Fifteen meaningful clusters were identified in total. According to Hsieh and Shannon (2005), 10 to 15 different clusters are eligible if the clusters are broad enough to cover many codes. In the *fifth step*, the meaningful clusters were interpreted for their meaning and categorised. Word was used for documentation throughout the process of analysis. An example of the analysis is presented in Table 2.

**TABLE 2:** Example of the analysis process.

Meaning unit	Condensed meaning unit	Code	Cluster	Subcategory
‘*she was so very believable, she cried and I cried…eh…they were good at their job* (acting as patient), *they acted just as if it was for real…I was moved to tears and kind of taken by her…when she opened up and told me what had happened’*.	The instructor's acting was true to life, she gave vent to emotions that also affected my emotions which made me cry.	Realistic performances that evoked emotion and compassion	Realistic acting	The simulation training was authentic and realistic

### 2.6 Ethical Considerations

This study was approved by the Council for Ethics at the Department of Nursing and Health at Gothenburg University. Participants were informed about the study orally and in writing. They were also informed that their participation was voluntary and could be terminated at any time. The information was repeated at the time of the intervention when written informed consent was collected. All data were coded and kept confidential.

### 2.7 Rigour and Reflexivity

Credibility was sought by using in‐depth interviews with semi‐structured open‐ended questions to capture participants’ experiences. To ensure trustworthiness, the first two authors analysed the data together in the first phase, following the approach described by Hsieh and Shannon (2005) to maintain an overall perspective. The focus of the analysis was on interpretation and meaning‐making to capture participants’ experiences. The first two authors worked together to reduce the codes to categories. In the fourth step, the analysis was discussed with the third author in relation to the categorisation. All three authors together reflected upon the categories in relation to transcribed verbatims and to the meaning of the codes. Credibility was pursued by the three authors contributing different perspectives in an open dialogue. Throughout the process, trustworthiness was sought by using a systematic and reflexive analysis. To validate the interpretation, categories are described and illustrated with quotes. Transferability was ensured by describing the study setting/context, recruitment and participants, the data collection, and the process of data analysis.

## 3 Results

**FIGURE 3:**
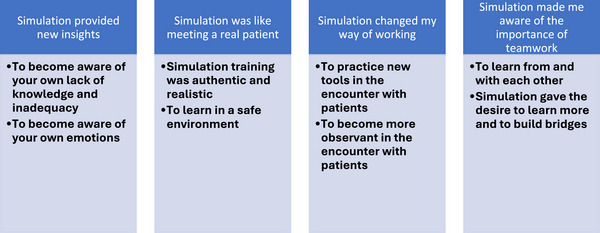
The result is presented in four categories and eight subcategories.

The results are presented in four categories and eight subcategories (Figure 3).

### 3.1 Simulation Provided New Insights

The training revealed gaps in participants' previous knowledge, leading to feelings of inadequacy and regret over not having asked past patients about IPV.

#### 3.1.1 To Become Aware of Your Own Lack of Knowledge and Inadequacy

The ambulance nurses experienced that simulation make them realise  their lack of knowledge about IPV. It raised questions about their own previous ability to identify victims of IPV and an awareness of how many patients they may have missed over the years due to their lack of knowledge.

How many people do we actually bump into without knowing, how tragic it is that it can be concealed to such an extent that we can't address it. There were many tragic feelings, like, how many victims are there really. And how many do we miss? (Interview 4)

The previous lack of knowledge led to feelings of inadequacy and a sort of paralysis during the simulation training. However, the feelings that emerged led to meaningful discussions during the debriefing and increased self‐awareness. The realisation of insufficient knowledge was a strong motivation to learn more.

#### 3.1.2 To Become Aware of Your Own Emotions

Participants experienced that they were affected by the seriousness of the scenarios in the simulation training, feeling sympathy and empathy for the victims. Emotions such as frustration, anger, sorrow, and hopelessness arose during the training and in the debriefing afterwards.

Feelings of frustration and hopelessness related to the victims of IPV blaming themselves, normalising and defending their perpetrator's behaviour. The victims’ ambivalence about leaving their perpetrator and the destructive relationship was also something that led to frustration among the ambulance nurses.

And it stirred up a lot of thoughts and emotions within oneself, especially frustration. Well, this, when you witness a situation that is so wrong… When someone is treated so badly, and you just want to remove them from the situation, but you can't because that person doesn't want it themselves, or they're afraid, or unable, or just… And they defend their perpetrator and all of this, I understand that it's like this, these mechanisms, but it becomes very, well, it becomes very, there's an internal battle while you're supposed to be professional and address her in her situation where she is, without, well, destroying everything. (Interview 5)

During the debriefing, it became clear that the participants were not alone in struggling with all the feelings within themselves. Nevertheless, towards the patient, they projected a sense of security, calm, and comfort. The participants also experienced comfort from knowing that good care can happen even if they are not able to resolve the situation completely.

### 3.2 Simulation Was Like Meeting a Real Patient

The simulation training was like meeting a real patient and made it possible to act authentically and realistically in the role of an ambulance nurse. The context was experienced as safe for learning, as no patient could be harmed.

#### 3.2.1 Simulation Training Was Authentic and Realistic

Persons acting as patients and their ability to mimic reality made it possible for the participants to react in their professional role as ambulance nurses. The acting out of emotional expressions was experienced as real and authentic and moved the participants to tears, even though the cases were not real.

But she was so incredibly credible, she cried and I cried… so they were really good at their job, it was as if it was real… I was moved to tears and like, touched by her… when she opened up and told me what had happened. (Interview 2)

Even if the simulated training session felt realistic, some participants experienced it as difficult to act on intuition because the cases were not real. Despite that, the overall experience of the simulation was that it was easy for the participants to act in a professional manner.

#### 3.2.2 To Learn in a Safe Environment

The simulation training was an opportunity to apply theoretical knowledge gained from web‐based education in a practical setting. The setting was experienced as a safe place to practise the encounter with victims of IPV, as any inadequate acting would not harm the patient. Having simulation training in smaller groups contributed to feeling safe.

Doing the simulation training with a work partner strengthened the feeling of being comfortable in daily work, allowing the ambulance nurses to truly see and converse with the patient on their own terms. Security in the working partnership also contributed to better care by creating security for the patient. Overall, good partnership was perceived as positive for the nurses’ individual and professional learning and growth.

### 3.3 Simulation Changed My Way of Working

The simulation training changed the participants’ way of working by providing new tools, giving them the courage to ask about potential victims being subjected to IPV. An increased awareness about interactions in the home environment led to them being more observant in their encounters with patients than before.

#### 3.3.1 To Practice New Tools in the Encounters With Patients

The ambulance nurses found simulation training with a focus on the encounter with a patient to be an unfamiliar experience, as they were more used to simulating acute and life‐threatening situations. Although the experience was unfamiliar and therefore difficult to cope with, it was still perceived as useful.

What we normally practise is very medically technical and following algorithms and such, but the encounter is just something we never practise, and it is, well, I think it is very useful. (Interview 5)

The participants found that the simulation training provided new tools that were useful in their clinical work. The tools focused on when, where, and how it could be appropriate to ask about exposure to violence, and there were also tools to get the patient to open up. This contributed to greater confidence in encountering victims of IPV by enhancing knowledge, awareness, and responsiveness. Fear and lack of knowledge were described as obstacles that prior to the simulation training stopped them from asking about violence. The new sense of confidence and having tools to facilitate making enquiries gave them the courage to ask questions about exposure to IPV.

I have asked almost all women […] How they have it at home… I have never asked this question before it is only now after the web‐based education and the simulation. (Interview 7)

The training increased their awareness and responsiveness not only as professionals but also in their close private settings. Knowledge and awareness made them more alert towards individuals also in their close private environment and within society at large. The participants said that the patients they asked about IPV gave positive feedback about asking, which encouraged them to continue to try to identify people living with violence.

One girl was very happy that the question arose, she had never been asked it before. She worked in healthcare herself, as a counsellor or psychologist. That response makes it easier to ask the question next time. And I just want to ask more questions. (Interview 7)

#### 3.3.2 To Become More Observant in the Encounter With Patients

The participants’ increased awareness about IPV through simulation training contributed to new observations in the patients' home environment. With increased attention to the interaction between patients and their relatives, a deeper understanding followed that there might be much more hidden behind what was said and observed.

(now) better awareness of it (IPV) … I have it (awareness of IPV) with me more often when I am at patients’… and you think a bit more of it when you are at some couple's home […] you reflect anyway how they interact. (Interview 5)

The increased awareness contributed to an increased interaction between colleagues about how to communicate with patients. Mutual thinking within the workplace had started to develop, including openness to discussing IPV and how to talk about it with patients.

### 3.4 Simulation Made Me Aware of the Importance of Teamwork

Learning together and from each other was experienced as a central part of the simulation training. The importance of working together in the team and constantly learning from each other became evident. A need for building bridges to other responsible actors while caring for victims subjected to IPV was expressed.

#### 3.4.1 To Learn From and With Each Other

Simulation training gave the nurses the opportunity to be inspired by observing colleagues while at the same time allowing for hands‐on practice. Observing each other provided insights into what makes a good encounter. Learning also came from encounters that did not turn out well, though it was perceived as more difficult.

We go around thinking we're great, but then we see ourselves on video, and ouch, it wasn't good at all. I believe many are unaware of how they actually encounter people. (Interview 3)

No matter whether the encounter was good or if it could be better, it was important to receive feedback and discuss encounters in daily work. This was not a priority before. The simulation training was an opportunity for the nurses to get to know themselves and their colleagues better. Debriefing on the simulation training contributed to a change in the personal way of thinking, raising self‐awareness.

#### 3.4.2 Simulation Gave the Desire to Learn More and to Build Bridges

The knowledge gained from simulation training was shared with colleagues. A desire to learn even more was fostered, along with a commitment to deepening knowledge about IPV in the workplace. The participants wanted to give all colleagues the opportunity to simulate encounters with people suspected of being victims of IPV.

During the simulation, the roles of other authorities in handling victims of IPV were highlighted. Recognising that healthcare professionals alone cannot assist those who are victims of IPV, the need to bridge gaps between ambulance services and other authorities such as the police and social services became evident.

## 4 Discussion

In this study, ambulance nurses' experiences of simulation related to IPV and how they perceive their work after completing the simulation were analysed. The results showed the importance of training in effective communication and teamwork among the participating ambulance nurses. The results also showed that simulations addressing IPV can open new doors through providing new knowledge and insights. The participants experienced strengthened confidence in asking about exposure to violence after the simulation training, which resulted in more patients being asked about this issue.

Simulation training about IPV was experienced by the participating ambulance nurses to increase their self‐awareness and self‐confidence when meeting a person exposed to IPV. By observing each other during the simulation and with the help of debriefing, the participants became more aware of their own actions and approaches in the meeting with a person exposed to IPV. Self‐awareness has been described by nurses as a process of identifying strengths and limitations that can increase nurses’ confidence in managing challenging and complex situations (Younas et al. 2019). Previous research has shown that nurses' self‐awareness can be improved by training that includes reflecting on personal behaviours, thoughts, and feelings and on the impact on clinical situations (Woroch and McNamara 2021; Han and Kim 2016). Woroch and McNamara (2021) showed that simulation training gave advanced practice nursing students increased confidence and preparedness to identify and address IPV clinically. Conversely, low rates of confidence, knowledge, and clinical skills negatively affect nurses’ preparedness to respond to IPV (Withiel et al. 2022, 2023; Alhalal 2020). Thus, the simulation that increases nurses' confidence and self‐awareness can have positive effects on responding to IPV.

To our knowledge, this study is the first to focus on ambulance nurses’ encounters with a patient suspected of being a victim of IPV. Previous research on simulation for nurses in ambulance services has focused on medical assessment and clinical decision‐making skills (Wallin et al. 2021). However, the findings from our study indicate that there might also be a need for simulation training with a focus on the encounter with the patient. Patients have described the importance of good communication and a compassionate approach in ambulance healthcare, since the best medical care and treatment can never compensate for the feeling of not being taken seriously or listened to (Norberg Boysen et al. 2017). Similarly, a review of patients’ complaints about ambulance operations found that the interactions with nurses were more important to patients than the level of medical competence demonstrated (Ahlenius et al. 2017). This is highly applicable for patients who might have been subjected to IPV, as their need for trust in the nurse is of particular importance. Hence, simulation training for ambulance nurses must focus not only on acute and medical areas but also on the encounter with the patient and especially on the encounter with potential victims of IPV.

Simulation about IPV can provide increased confidence in the working partnership within ambulance healthcare. Joint education through simulation can enhance understanding and skills in communication, which can contribute to the team gaining increased confidence and establishing common strategies (Alharbi et al. 2024; Shrivastava et al. 2022). Nurses felt that simulation provided them with collegial confidence in communication, improved interaction, and gave an opportunity to discuss and develop common strategies for the encounter. Thus, it appears that simulation involving communication increases knowledge and provides increased confidence within a team, which can be of great importance in healthcare to generate a safer encounter with persons who have been subjected to IPV.

## 5 Limitations

Some limitations are worth noting. This study was limited by the small sample size, with only seven participants. The intervention and the data collection were limited to one ambulance service, only covering a portion of the ambulance nurses. Although the interviews were relatively short, we believe that they each contributed rich data. We found no correlation between interview length and the amount of data provided. This may be partly explained by the time participants had between the intervention and the interview, as scheduling the interviews at the time of the intervention allowed them time to reflect on their experiences. However, more participants and interviews would have the potential to add and deepen data about nurses’ experiences of the intervention. Additional studies are needed to further investigate the potential of using simulation for training about IPV both in the context of ambulance services and in other contexts. Follow‐up on a longer time scale than seven to nine weeks after the intervention is also warranted.

## 6 Conclusions

Simulating situations involving interpersonal interactions and IPV might help ambulance nurses increase their self‐awareness and gain practical tools to handle complex situations. Simulation training and increased knowledge can give nurses confidence and security, which in turn can enhance patient safety by allowing nurses to more effectively identify and provide support to individuals experiencing IPV.

### 6.1 Implications for Nursing and Health Policy

The focus of this study was ambulance nurses. However, the findings might also be applicable to nurses in other contexts. District nurses in primary care and nurses in home‐based care work can have similar encounters with their patients as ambulance nurses. Training through online courses and simulations can increase knowledge and preparedness among clinically active nurses about IPV. It can also give nurses greater belief in their own ability to identify IPV. Consequently, this can lead to nurses more frequently asking about IPV. Increased awareness among nurses about their role in encounters with individuals who are victims of IPV is one aspect of addressing this global challenge facing public health.

## Author Contributions

Study design: EEM, JJ, and KS. Data collection: EEM and JJ. Data analysis: EEM and JJ. Study supervision: KS. Manuscript writing: EEM, JJ, and KS. Critical revisions for important intellectual content: EEM, JJ, and KS.

## Conflicts of Interest

The authors have no conflicts of interest to declare.

## References

[inr70030-bib-0001] Ahmad, I. , P. A. Ali , S. Rehman , A. Talpur , and K. Dhingra . 2017. “Intimate Partner Violence Screening in Emergency Department: A Rapid Review of the Literature.” Journal of Clinical Nursing 26, no. 21–22: 3271–3285. 10.1111/jocn.13706. 28029719

[inr70030-bib-0002] Alhalal, E. 2020. “Nurses' Knowledge, Attitudes and Preparedness to Manage Women With Intimate Partner Violence.” International Nursing Review 67, no. 2: 265–274. 10.1111/inr.12584. 32301110

[inr70030-bib-0003] Ahlenius, M. , V. Lindström , and V. Vicente . 2017. “Patient's Experience of Being Badly Treated in the Ambulance Service: A Qualitative Study of Deviation Reports in Sweden.” International Emergency Nursing 30: 25–30. 10.1016/j.ienj.2016.07.004. 27567212

[inr70030-bib-0004] Alshammari, A. , C. Evans , and J. Mcgarry . 2023. “Nurses' Experiences of Perceiving Violence and Abuse of Women in Saudi Arabia: A Phenomenological Study.” International Nursing Review 70, no. 4: 501–509. 10.1111/inr.12859. 37401925

[inr70030-bib-0005] Alharbi, A. , A. Nurfianti , R. F. Mullen , J. D. McClure , and W. H. Miller . 2024. “The Effectiveness of Simulation‐Based Learning (SBL) on Students' Knowledge and Skills in Nursing Programs: A Systematic Review.” BMC Medical Education 24, no. 1: 1099. 10.1186/s12909-024-06080-z. 39375684 PMC11459713

[inr70030-bib-0006] Allison, A. , A. Weerahandi , T. Johnson , et al. 2023. “A Scoping Review on the Use of Experiential Learning in Professional Education on Intimate Partner Violence.” Journal of Family Violence 4: 1–20. 10.1007/s10896-023-00552-4. PMC10157572 37358988

[inr70030-bib-0007] Andersson, U. , H. Maurin Söderholm , B. Wireklint Sundström , M. Andersson Hagiwara , and H. Andersson . 2019. “Clinical Reasoning in the Emergency Medical Services: An Integrative Review.” Scandinavian Journal of Trauma, Resuscitation and Emergency Medicine 27, no. 1: 76. 10.1186/s13049-019-0646-y. 31426839 PMC6700770

[inr70030-bib-0008] Aregger Lundh, A. , C. Tannlund , and A. Ekwall . 2022. “More Support, Knowledge and Awareness Are Needed to Prepare Emergency Department Nurses to Approach Potential Intimate Partner Violence Victims.” Scandinavian Journal of Caring Sciences 37: 397–405. 10.1111/scs.13123. 36114694

[inr70030-bib-0010] Cunningham, S. , C. Cunningham , and L. Foote . 2020. “Recognizing Elder Abuse: An Interprofessional Simulation Experience With Prelicensure Health Care Students.” Journal of Geriatric Physical Therapy 43, no. 4: E58–E64. 10.1519/JPT.0000000000000257. 31913215

[inr70030-bib-0011] Han, S. , and S. Kim . 2016. “An Integrative Literature Review on Self‐Awareness Education/Training Programs in the Nursing Area.” Perspectives in Nursing Science 13, no. 2: 59. 10.16952/pns.2016.13.2.59.

[inr70030-bib-0013] Hinsliff‐Smith, K. , and J. McGarry . 2017. “Understanding Management and Support for Domestic Violence and Abuse Within Emergency Departments: A Systematic Literature Review From 2000–2015.” Journal of Clinical Nursing 26, no. 23–24: 4013–4027. 10.1111/jocn.13849. 28403521

[inr70030-bib-0014] Hsieh, H. , and S. Shannon . 2005. “Three Approaches to Qualitative Content Analysis.” Qualitative Health Research 15, no. 9: 1277–1288. 16204405 10.1177/1049732305276687

[inr70030-bib-0015] Johnson, P. , and M. Montgomery . 2017. “Improving Nursing Students Comfort Dealing With Intimate Partner Violence.” Teaching and Learning in Nursing 12: 286–288.

[inr70030-bib-0016] Lee, P.‐Y. , and B.‐O. Lee . 2022. “Effectiveness of Simulation‐Based Education on Nursing Students' Professional Knowledge, Attitude and Self‐Confidence in Handling Child Abuse Cases.” Nurse Education in Practice 65: 103480. 10.1016/j.nepr.2022.103480. 36327597

[inr70030-bib-0017] Lindström, V. , K. Bohm , and L. Kurland . 2015. “Prehospital Care in Sweden.” Notfall Rettungsmedizin 18: 107–109. 10.1007/s10049-015-1989-1.

[inr70030-bib-0018] Miller, C. J. , O. L. Adjognon , J. E. Brady , M. E. Dichter , and K. M. Iverson . 2021. “Screening for Intimate Partner Violence in Healthcare Settings: An Implementation‐Oriented Systematic Review.” Implementation Research and Practice 2: 26334895211039894. 10.1177/26334895211039894. 36712586 PMC9881185

[inr70030-bib-0019] NCK (The National Center for Knowledge on Men's Violence Against Women) . 2022. Bemötande och behandling av personer som utsatts för våld. Uppsala: Nationellt centrum för kvinnofrid (in Swedish).

[inr70030-bib-0041] Norberg Boysen, G. , Nyström, M. , Christensson, L. , Herlitz, J. , and Wireklint Sundström, B. 2017. “Thrust in the Early Chain of Healthcare: Lifeworld Hermeneutics From the Patient's Perspective.” International Journal of Qualitative Studies on Health and Well‐Being 12, no. 1: 1748–2631. 10.1080/17482631.2017.1356674. PMC5590623 28793852

[inr70030-bib-0040] Öhman, A. , C. Vives‐Cases , and K. Edin . 2024. “Important, but Difficult': Swedish Primary Care Professionals' Perceptions and Experiences of Dealing With Violence Against Women: An Interview Study.” BMC Primary Care 25, no. 1: 258. 10.1186/s12875-024-02489-z. 39014330 PMC11251211

[inr70030-bib-0022] O'Rourke, L. A. , M. Morrison , A. Grimsley , and V. T. Cotter . 2023. “High‐Fidelity Simulation and Nurse Clinical Competence: An Integrative Review.” Journal of Clinical Nursing 32, no. 9–10: 1549–1555. 10.1111/jocn.16028. 34453385

[inr70030-bib-0023] Price, A. , and K. Couch . 2023. “Patient‐Centered Intimate Partner Violence Screening, Brief Intervention, and Referral to Treatment.” Nursing for Women's Health 27, no. 4: 291–300. 10.1016/j.nwh.2023.02.005. 37321558

[inr70030-bib-0024] Purwaningtyas, N. , G. Wiwaha , E. Pudji‐Setiawati , and I. Desy‐Arya . 2019. “The Role of Primary Healthcare Physicians in Violence Against Women Intervention Program in Indonesia.” BMC Family Practice 20: 168. 10.1186/s12875-019-1054-0. 31801466 PMC6892181

[inr70030-bib-0025] Ross, M. E. T. , J. Bryan , K. Thomas , A. Asghar Ali , and S. Pickens . 2020. “Elder Abuse Education Using Standardized Patient Simulation in an Undergraduate Nursing Program.” Journal of Nursing Education 59, no. 6: 331–335. 10.3928/01484834-20200520-06. 32497235

[inr70030-bib-0026] Sammut, D. , J. Kuruppu , K. Hegarty , and C. Bradbury‐Jones . 2021. “Which Violence Against Women Educational Strategies Are Effective for Prequalifying Health‐Care Students?: A Systematic Review.” Trauma, Violence & Abuse 22, no. 2: 339–358. 10.1177/1524838019843198. 31122182

[inr70030-bib-0027] Shrivastava, S. , J. Martinez , D. J. Coletti , and A. Fornari . 2022. “Interprofessional Leadership Development: Role of Emotional Intelligence and Communication Skills Training.” MedEdPORTAL 18: 11247. 10.15766/mep_2374-8265.11247. 35634034 PMC9098732

[inr70030-bib-0028] Sprague, S. , K. Madden , N. Simunovic , et al. 2012. “Barriers to Screening for Intimate Partner Violence.” Women's Health 52, no. 6: 587–605. 10.1080/03630242.2012.690840. 22860705

[inr70030-bib-0029] Swedish National Board of Health and Welfare . 2022. “The National Board of Health and Welfare´s Investigations Into Certain Injuries and Deaths 2018–2021.” In Socialstyrelsens utredningar av vissa skador och dödsfall 2018–2021 (in Swedish). Socialstyrelsen. Socialstyrelsens utredningar av vissa skador och dödsfall 2018–2021 (accessed 12 June 2024).

[inr70030-bib-0030] Swedish National Board of Health and Welfare . 2023. “Sweden's Pre‐Hospital Emergency Care: Current Picture, Assessment and Development Proposals.” In Sveriges Prehospitala Akutsjukvård‐ Nulägesbild, Bedömning Och Utvecklingsförslag (in Swedish). Socialstyrelsen. Sveriges prehospitala akutsjukvård (socialstyrelsen.se) (accessed 12 June 2024).

[inr70030-bib-0031] Sundborg, E. , L. Törnkvist , N. Saleh‐Stattin , P. Wändell , and I. Hylander . 2015. “To Ask, or Not to Ask: The Hesitation Process Described by District Nurses Encountering Women Exposed to Intimate Partner Violence.” Journal of Clinical Nursing 26, no. 15–16: 2256–2265. 10.1111/jocn.12992. 26419327

[inr70030-bib-0032] Wallin, K. , C. Werkander Harstäde , A. Bremer , and U. Hörberg . 2021. “Nurse Preceptors' Experience‐Based Strategies for Supporting Learning in the Ambulance Service: A Combined Focus Group and Dyadic Interview Study.” Journal of Advanced Nursing 78: 1704–1717. 10.1111/jan.15127. 34873737

[inr70030-bib-0033] Withiel, T. D. , H. Gill , and C. A. Fisher . 2021. “Responding to Family Violence: Variations in Knowledge, Confidence and Skills Across Clinical Professions in a Large Tertiary Public Hospital.” SAGE Open Medicine. 9: 20503121211000923. 10.1177/20503121211000923. 33786184 PMC7958155

[inr70030-bib-0034] Withiel, T. D. , S. Sheridan , N. Rudd , and C. A. Fisher . 2022. “Preparedness to Respond to Family Violence: A Cross‐Sectional Study across Clinical Areas.” SAGE Open Nursing. 8: 23779608221126355. 10.1177/23779608221126355. 36245850 PMC9557861

[inr70030-bib-0035] Withiel, T. D. , S. Sheridan , C. Rushan , and C. A. Fisher . 2023. “Multifaceted Training and Readiness to Respond to Family Violence: A Prospective Cohort Evaluation.” Journal of Clinical Nursing. 32, no. 21‐22: 7740–7750. 10.1111/jocn.16827. 37477159

[inr70030-bib-0036] Woroch, R. A. , and M. McNamara . 2021. “Intimate Partner Violence Standardized Patient Simulation for Nurse Practitioner Students.” Journal of the American Psychiatric Nurses Association 29, no. 4: 338–343. 10.1177/10783903211023557. 34151627

[inr70030-bib-0037] WHO (World Health Organization) . 2017. “Violence Against Women.” Accessed 12 June 12, 2024. https://www.who.int/news‐room/fact‐sheets/detail/violence‐against‐women.

[inr70030-bib-0038] WHO . 2013. Responding to Intimate Partner Violence and Sexual Violence Against Women: WHO Clinical and Policy Guidelines. World Health Organization. 24354041

[inr70030-bib-0039] Younas, A. , S. P. Rasheed , A. Sundus , and S. Inayat . 2019. “Nurses' Perspectives of Self‐Awareness in Nursing Practice: A Descriptive Qualitative Study.” Nursing & Health Sciences 22, no. 2: 398–405. 10.1111/nhs.12671. 31837204

